# Monthly Summary

**Published:** 1890-03

**Authors:** 


### Monthly Summary.

ARSENIC.—Dr. Kirk: This is a bug-a-boo that comes up periodically. From my earliest attendance at associations similar discussions have come up for and against arsenic, and still we continue to use it. I think the most unsatisfactory period of my professional life was about three years' time, when I discarded arsenic. I was afraid to use it. I was afraid somebody would find out that I used it, and would turn me out of the societies. I have placed arsenic in a tooth, and had no other plug but sandaric and cotton filling, and it remained there for three years. When he came back I took out the plug and found arsenic on the cotton. I cleaned out the pulp chamber and canals and filled them and the crowns, and no trouble came of it; a good success. I remember of one case where the death of the patient was supposed to have been caused by arsenic in a tooth; and yet it was proven that no arsenic was ever in the tooth. In the use of arsenic, I believe with Dr. Reid we should be careful in the cases. When a crown cavity exposes the pulp the precaution is limited, but the proximal cavities are the ones where extra care is required. —*Indiana Transactions.*

WHITE CHLORA-PERCHA.—Dr. Patterson thought the subject of root filling the most important before the profession to-day. He desired to direct attention to an improved solution of gutta-percha for filling root canals. The great majority of operators use red base-plate gutta-percha; but a much better solution is made with the white gutta-percha filling material, which has less shrinkage than the red. In crooked canals he uses this solution almost exclusively, sealing the apex, and filling the apical third or fourth of the canal. It is bad practice to force the filling material beyond the apex. Oxychloride of zinc is the best filling for the remainder of the canal. He finds no trouble in forcing it to any part of the tooth, and he then feels that the organic material is placed in a better condition than by any other method. Roots filled with gutta-percha, when cut open, have more odor than those filled with oxychloride.—*Archives of Dentistry.*

